# The value of primary and adjuvant radiotherapy for cutaneous squamous cell carcinomas of the head-and-neck region in the elderly

**DOI:** 10.1186/s13014-021-01832-3

**Published:** 2021-06-12

**Authors:** Erik Haehl, Alexander Rühle, Rabea Klink, Tobias Kalckreuth, Tanja Sprave, Eleni Gkika, Constantinos Zamboglou, Frank Meiß, Anca-Ligia Grosu, Nils H. Nicolay

**Affiliations:** 1 Department of Radiation Oncology, University of Freiburg - Medical Center, Robert-Koch-Str. 3, 79106 Freiburg, Germany; 2 German Cancer Research Center (Dkfz), German Cancer Consortium (DKTK) Partner Site Freiburg, Neuenheimer Feld 280, 69120 Heidelberg, Germany; 3 Department of Dermatology and Venereology, University of Freiburg - Medical Center, Hauptstr. 7, 79104 Freiburg, Germany

**Keywords:** Cutaneous squamous cell carcinoma, Head-and-neck tumor, Radiotherapy, Elderly, Geriatric

## Abstract

**Purpose:**

To examine treatment patterns, oncological outcomes and toxicity rates in elderly patients receiving radiotherapy for cutaneous squamous cell carcinoma (cSCC) of the head-and-neck region.

**Material and methods:**

In this retrospective single-center analysis, locoregional control (LRC), progression-free survival (PFS) and overall survival (OS) of elderly patients > 65 years with cSCC of the head-and-neck region undergoing radiotherapy between 2010 and 2019 were calculated. The prognostic value of clinicopathological parameters on radiotherapy outcomes was analyzed using the Cox proportional hazards model. In addition, both acute and chronic toxicities were retrospectively quantified according to CTCAE version 5.0.

**Results:**

A total of 69 elderly patients with cSCC of the head-and-neck region with a median age of 85 years were included in this analysis, of whom 21.7% (15 patients) presented with nodal disease. The majority of patients exhibited a good performance status, indicated by a median Karnofsky performance status (KPS) and Charlson Comorbidity Index (CCI) of 80% and 6 points, respectively. Radiotherapy was administered as primary (48%), adjuvant (32%) or palliative therapy (20%). 55 patients (79.7%) completed treatment and received the scheduled radiotherapy dose. Median EQD2 radiation doses were 58.4 Gy, 60 Gy and 51.3 Gy in the definitive, adjuvant and palliative situation, respectively. 2-year LRC, PFS and OS ranged at 54.2%, 33.5 and 40.7%, respectively. Survival differed significantly between age groups with a median OS of 20 vs. 12 months (*p* < 0.05) for patients aged 65–80 or above 80 years. In the multivariate analysis, positive lymph node status remained the only significant prognostic factor deteriorating OS (HR 3.73, CI 1.54–9.03, *p* < 0.01). Interestingly, neither KPS nor CCI impaired survival in this elderly patient cohort. Only 3 patients (4.3%) experienced acute CTCAE grade 3 toxicities, and no chronic CTCAE grade 2–5 toxicities were observed in our cohort.

**Conclusion:**

Radiotherapy was feasible and well-tolerated in this distinct population, showing the general feasibility of radiotherapy for cSCC of the head-and-neck region also in the older and oldest olds. The very mild toxicities may allow for moderate dose escalation to improve LRC.

**Supplementary Information:**

The online version contains supplementary material available at 10.1186/s13014-021-01832-3.

## Introduction

Non-melanoma skin cancers (NMSC) are among the five most frequent cancer entities with about 3 million new cases globally per year [1, 2]. Reported incidences vary widely according to ethnicity, geographic origin and age. With the lack of systematic coverage of NMSC in most cancer registers, incidence is likely underestimated [3]. Cutaneous squamous cell carcinomas (cSCC) account for around 20% of NMSCs and most often affect elderly patients. A large cohort study of cSCC patients reported a mean age at diagnosis of 70 years and the highest incidence in patients aged above 80 years [4]. With the ongoing demographic changes, the number of patients presenting with cSCC has been increasing rapidly [5]. Apart from rare genetic disorders such as xeroderma pigmentosum, overexposure to UV-light, immunosuppression and chronic scarring constitute major risk factors for cSCC. Wide local surgical excision as the current treatment standard provides excellent cure rates for the majority of cases, while curettage or cryotherapy are alternative treatments that result in similar patient outcomes for small and well-defined cSCCs [6–8]. Nodal or distant metastases develop rarely yet being the main reason for a disease-specific 5-year-mortality of around 2% [9, 10]. Increased metastatic risk is reported for deep infiltration, perineural invasion and chronic scarring [11–15].

However, the vast majority of cSCCs present in the head-and-neck region, in which wide local excisions harbor the risk for permanent mutilation [4, 7]. Additionally, distinct facial tumor subsites such as the oral lip and the ear are associated with significantly higher rates of nodal metastases of up to 10% and therefore more often require multimodal treatment strategies including radiotherapy [11, 12, 16]. Radiotherapy constitutes a curative treatment option if wide local excision is not possible or declined by the patient, and the addition of adjuvant radiotherapy to surgical treatments improves patient outcomes in case of lymph node involvement [12, 17, 18]. However, the benefit of adjuvant radiotherapy for high-risk tumor features such as perineural invasion remains controversial [18, 19].

Although cSCC is a disease of the elderly patient, there are only few studies that investigated the role of age regarding treatment outcomes. The newly published American Society for Radiation Oncology (ASTRO) guideline emphasizes the role of radiotherapy in the treatment of cSCC but gives no particular recommendation for the treatment of the elderly, probably due to the lack of evidence [20]. The present analysis seeks to contribute to closing this gap. In this single-center study, we analyzed demographic data, oncologic outcomes and toxicity rates of elderly patients receiving radiotherapy for cSCC between 2010 and 2019 at a major tertiary cancer center. In addition, risk factors correlating with treatment response were investigated in elderly cSCC patients.

## Material and methods

### Patients and treatment

This retrospective single-center analysis enrolled all patients older than 65 years treated with radiotherapy for histologically confirmed cSCC of the head-and-neck region between 2010 and 2019 at the Department of Radiation Oncology, University of Freiburg Medical Center. The study was approved in advance by the institutional ethical review committee (reference no. 551/18). Demographic and clinical data were retrospectively collected from electronic patient files, and pathological data were extracted from the pathology reports.

**Fig. 1:**
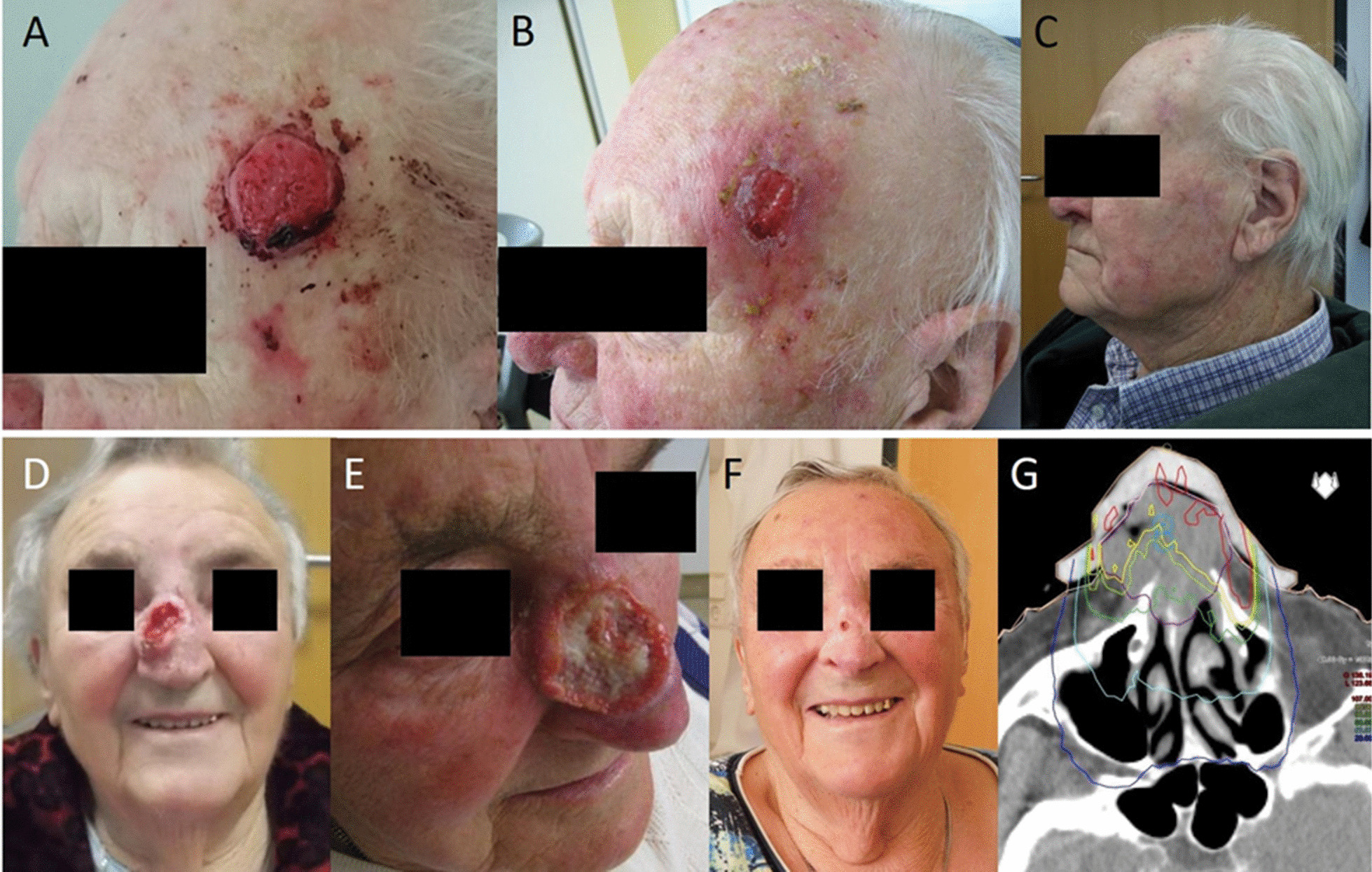
Representative images of a 94-year old patient with cSCC of the left temple before (**A**), at the end (**B**) and 6 weeks after (**C**) electron beam radiotherapy with 51 Gy-. 88-year old patient with cSCC of the nose before (**D**), at the end (**E**) and 5 months after (**F**) electron beam radiotherapy with 48 Gy. (**G**) depictures the dose distribution of the electron field

Treatment for all patients was based on multidisciplinary tumor board recommendations. For photon radiotherapy, patients were immobilized with individually molded thermoplastic masks. Radiotherapy planning was conducted with Oncentra MasterPlan® (Nucletron BV, Veenendaal, The Netherlands) and Eclipse™ planning softwares (Varian Medical Systems). Depending on the target volume, conformal 3-dimensional radiotherapy (3DRT), intensity-modulated radiotherapy (IMRT) or linear accelerator-generated electron beam radiotherapy were used for treatment (Fig. 1).

### Survival and toxicity assessment

All patients were scheduled for routine follow-up examination at 3 months after radiotherapy and annually thereafter. Additional dermatological follow-up took place in 6-monthly intervals. In case of clinical evidence for local/locoregional recurrence or distant metastases, follow-up imaging examinations were carried out at the discretion of the treating physician. Overall survival (OS) was calculated from the completion of treatment to death from any cause, and progression-free survival (PFS) was assessed as the interval between treatment completion and disease progression at any site or death of any cause. Locoregional control (LRC) was defined as the absence of any progression of the primary tumor or the onset or progression of any cervical lymph node metastases. Missing survival data were acquired from the record sections of the federal state authorities of Baden-Württemberg through the Comprehensive Cancer Center Freiburg. Acute and chronic toxicities were classified based on the Common Terminology Criteria for Adverse Events (CTCAE), version 5.0. To assess the burden of comorbidity, the age-adjusted Charlson Comorbidity Index (CCI) was used in a modified version with no points given for cSCC itself.

### Statistical analyses

Actuarial OS, PFS and LRC rates were analyzed using the Kaplan–Meier method with the log-rank test to evaluate statistical significance. Univariate and multivariate analyses were performed using the Cox proportional hazards model. *P*-values below 0.05 were considered statistically significant. All statistical analyses were carried out using IBM SPSS Statistics software version 27 (IBM, Armonk, NY, USA).

## Results

### Patient and treatment characteristics

A total of 69 patients aged 65 years and above with histologically confirmed cSCCs of the head-and-neck region were included in this analysis (Table 1). The most common tumor localizations were nose, ear and cheek (n = 15, 14 and 9; Additional file 1: Table S1). Median patient age amounted to 85 years (range 66–99 years). Overall patient performance status was satisfactory in this elderly patient cohort with a median Karnofsky performance status (KPS) of 80% (range 40–100%) and 80% of patients having a KPS status of 70% or higher. Comorbidity burden was moderate with a median score of 6 (range 2–10) in the modified age-adjusted CCI, considering advanced patient age.

**Table 1:** Patient characteristics of elderly patients with cSCC of the head-and-neck region treated with radiotherapy between 2010 and 2019 (n = 69)

	n	%
**Sex**		
Male	39	56.5
Female	30	43.5
**Age**		
mean (range)	84	66–99
65–80	20	29.0
> 80	49	71.0
**Presentation**		
Initial diagnosis	26	37.7
Local recurrence	30	43.5
Nodal recurrence	13	18.8
**T stage**		
T1	11	15.9
T2	6	8.7
T3	14	20.3
T4	5	7.2
n/a	33	47.83
**N stage**		
N0	23	33.3
N1	4	5.8
N2	10	14.5
N3	1	1.4
n/a	31	44.9
**M stage**		
M0	23	33.3
M1	4	5.8
Mx	6	8.7
n/a	36	52.2
**Grading**		
G1	6	8.7
G2	35	50.7
G3	19	27.5
n/a	9	13.0
**R-status (if adjuvant)**		
R0	5	22.7
Rx	3	13.6
R1	10	45.5
R2	2	9.1
n/a	2	9.1
**KPS**		
Median (range)	80%	(40–100%)
100–90%	17	24.6
80–70%	38	55.0
60–50%	8	11.6
< 50%	3	4.3
n/a	3	4.3
**CCI**		
Median (range)	6	(2–10)
≤ 4	16	23.2
5	15	21.7
6	19	27.5
7	17	24.6
≥ 8	2	2.9

*KPS* Karnofsky performance status, *CCI* Charlson Comorbidity Index

The main reason for referral to radiotherapy was local recurrent disease after primary treatment in 43.5% (n = 30) of cases, followed by primary radiotherapy at initial diagnosis (n = 26, 37.7%) and metachronous nodal recurrence of cSCC (n = 13, 18.8%). 15 patients (21.7%) had clinical lymph node involvement, and only 4 patients (5.8%) presented with distant metastases. 22 patients (31.9%) were treated with postoperative radiotherapy after primary resection, mostly due to positive resection margins or remaining tumor (n = 15, 68%).

Radiotherapy was administered in curative intent in 55 patients (79.7%) and as palliative treatment in 14 patients (20.3%) (Table 2; Additional file 1: Table S2). Photon radiotherapy was the main treatment modality for 46 patients (66.7%); 19 patients (27.5%) were treated with electron beam radiotherapy and 4 patients (5.8%) received mixed-beam radiotherapy. Integrated and sequential boosts were used in 7 (10.1%) and 18 (26.1%) patients, respectively. Median administered radiation doses (EQD2) were 58.4 Gy, 60 Gy and 51.3 Gy in the definitive, adjuvant and palliative setting, respectively. Initially scheduled radiation doses for the primary and adjuvant setting were both 60 Gy. Fractionation regimes were heterogeneous: 59.4–70 Gy in conventional fractionation was the most frequently used (n = 35, 50.7%), hypofractionation with 11–13 fractions of 4 Gy was used in 10 cases (14.5%) (Additional file 1: Table S3). In 27 patients (39.1%), therapy comprised elective nodal irradiation. 55 patients (79.7%) completed the scheduled radiotherapy. Non-completion of radiotherapy was mostly due to treatment-related toxicities (n = 7; Additional file 1: Table S4), comorbidity (n = 3) and disease progression (n = 2) during radiotherapy. Only one patient received concomitant chemotherapy with mitomycin C and 5-fluorouracil. Median time to the last visit in our clinic was 8 months. Median follow-up calculated with the reversed Kaplan–Meier method for OS was 44 months.

**Table 2:** Treatment details for radiotherapy of elderly cSCC patients (n = 69)

	n	%
**Radiotherapy**		
Primary	33	47.8
Adjuvant	22	31.9
Palliative	14	20.3
Photons	46	66.7
Electrons	19	27.5
Both	4	5.8
Boost	25	36.2
Integrated	7	10.1
Sequential	18	26.1
Radiotherapy completed	55	79.7
Radiotherapy discontinued	14	20.3
**Definitive radiotherapy**		
Median radiation dose (EQD2)		58.4 Gy
Median single dose (EQD2)		2 Gy
Radiotherapy completed		85%
**Adjuvant radiotherapy**		
Median radiation dose (EQD2)		60 Gy
Median single dose (EQD2)		2 Gy
Radiotherapy completed		77%
**Palliative radiotherapy**		
Median radiation dose (EQD2)		51.3 Gy
Median single dose (EQD2)		2.75 Gy
Radiotherapy completed		71%
**Reason for non-completion**		
Tumor progress		2
Toxicity		7
Comorbidities		3
Patient request		2

### Treatment outcome

**Fig. 2:**
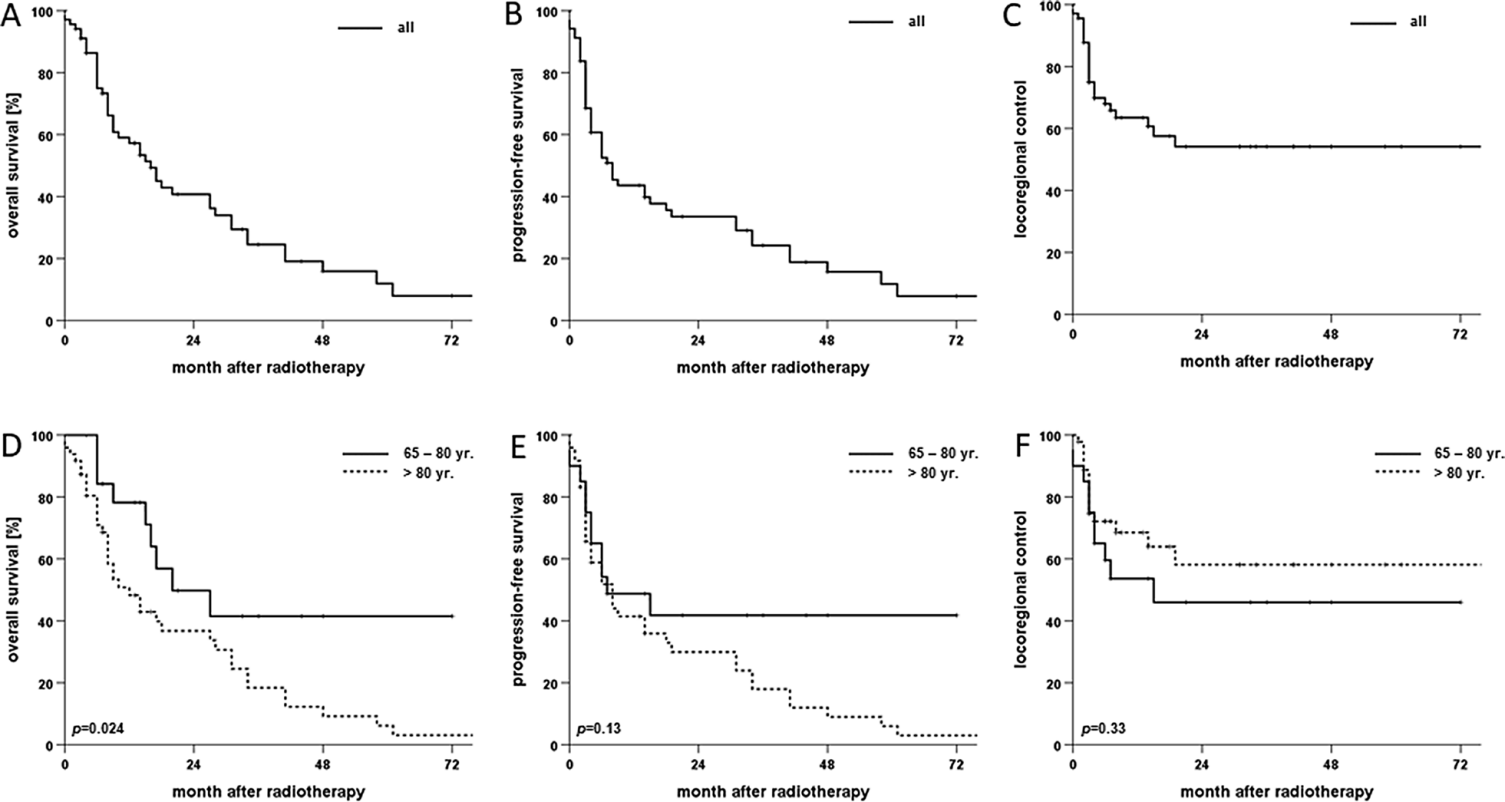
Kaplan–Meier curves for OS, PFS and LRC of elderly cSCC patients (> 65 years) following radiotherapy (n = 69) for the complete cohort (**A**–**C**) and in dependence of age separated at 80 years (**D**–**F**), respectively. *P*-values of log-rank tests are shown

**Fig. 3:**
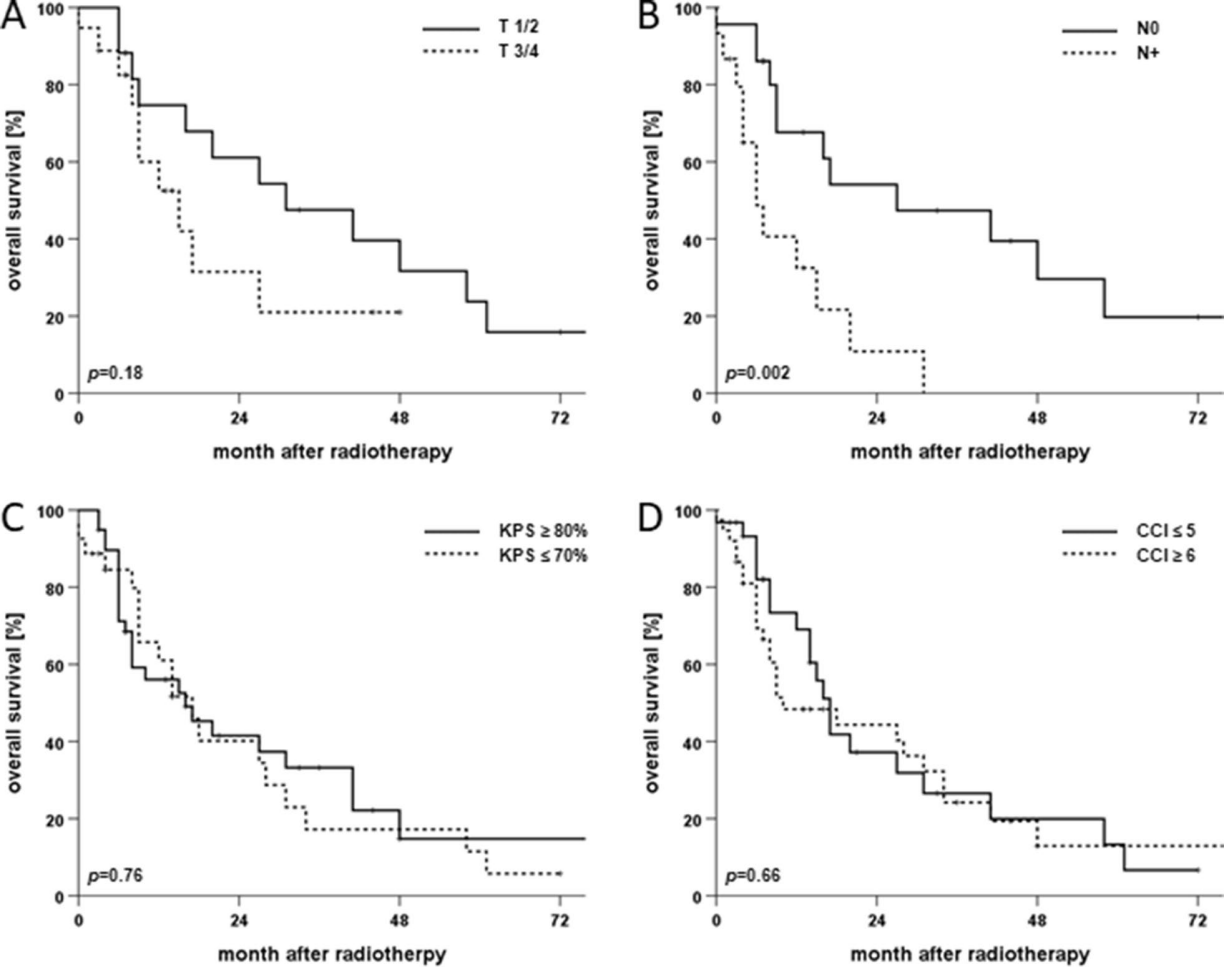
Kaplan–Meier curves for OS of elderly cSCC patients (> 65 years) after radiotherapy in dependence of T stage (**A**), N Stage (**B**), KPS (**C**) and modified CCI (**D**), n = 35, 37, 66 and 69, respectively. *P*-values of log-rank tests are displayed

**Fig. 4:**
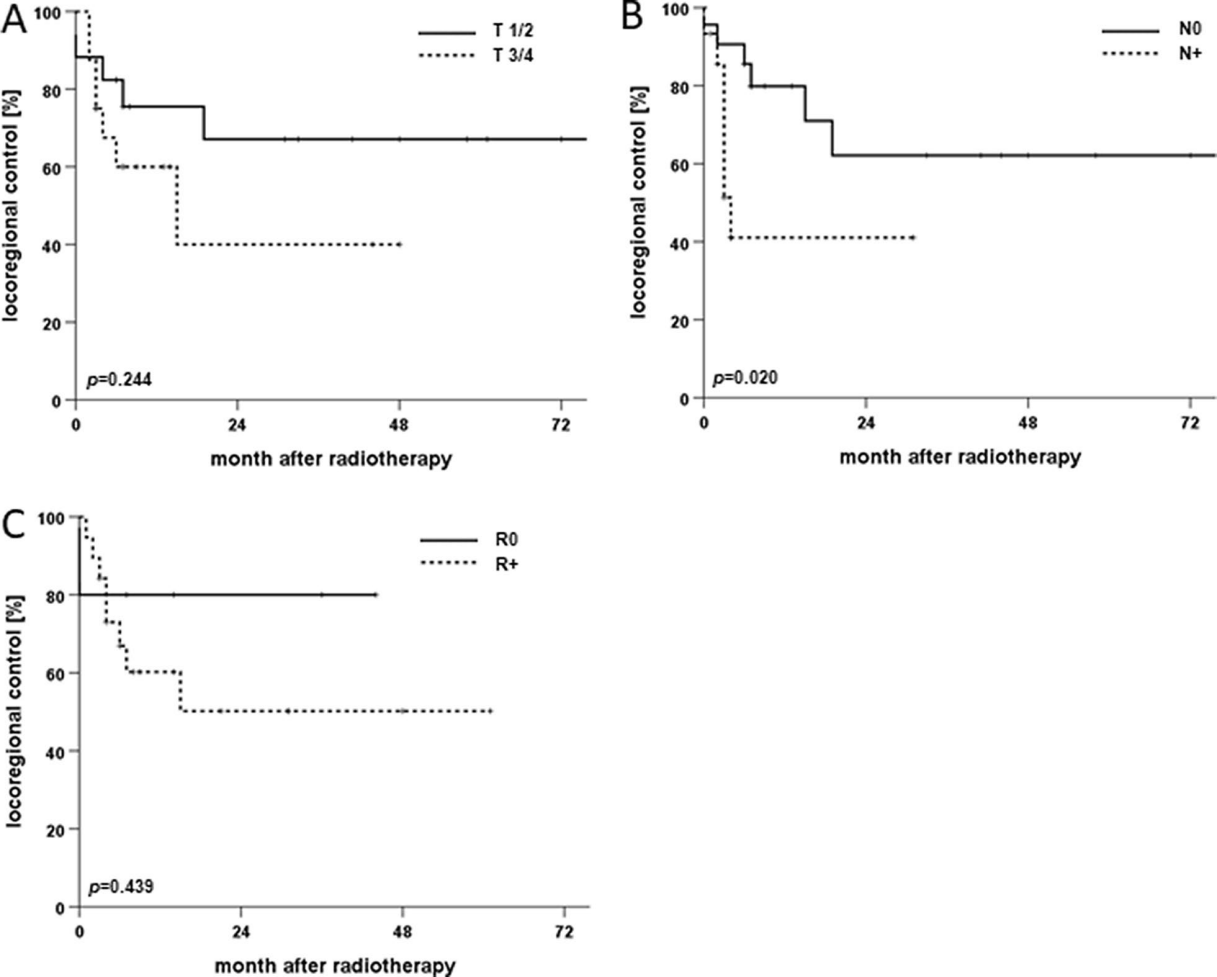
Kaplan–Meier curves for LRC of elderly cSCC patients (> 65 years) following radiotherapy in dependence of T stage (**A**), N Stage (**B**) and resection margin (**C**), n = 35, 37 and 26, respectively. *P* values of log-rank-tests are displayed

For the whole patient cohort, 2-year rates for OS, PFS and LRC amounted to 40.7%, 33.5% and 54.2%, respectively (Fig. 2). Median OS and PFS were 16 and 8 months, respectively, while median LRC was not reached. 25 (36.2%) patients experienced locoregional recurrence after therapy, 18 (26.1%) at the primary tumor siteand 15 (21.7%) as nodal recurrence (8 (11.6%) patients experienced both local and nodal recurrence). Survival differed significantly between age groups with a median OS of 20 months in patients aged 65 to 80 years compared to only 12 months in patients above 80 years (*p* < 0.05, log-rank test). Median PFS was comparable among all age groups and ranged at 8 months for patients up to 80 years versus 7 months for patients older than 80 years (*p* = 0.13). Similarly, LRC did not differ significantly between age groups (*p* = 0.33). Of the analyzed parameters, lymph node involvement had the strongest influence on survival with a median OS of 6 (N+) and 27 months (N0), respectively (*p* < 0.01) (Fig. 3). The prognostic value of nodal involvement was found strongest for the subgroup of patients older than 80 years with a median OS of 34 versus 8 months (*p* < 0.01). For patients aged 65 to 80 years, the negative influence of nodal involvement was not statistically significant, probably due to the small sample size (*p* = 0.314). Positive resection margins prior to radiotherapy were shown to result in a trend towards decreased OS (*p* = 0.06), while T stage, low patient performance or a higher comorbidity burden did not significantly influence OS (*p* = 0.18, *p* = 0.76 and *p* = 0.66, respectively). T stage (*p* = 0.422) and resection margin (*p* = 0.439) did not impact LRC, whereas lymphonodal spread was found to significantly deteriorate LRC in the Kaplan-Meier analyses (*p* < 0.05) (Fig. 4).

**Fig. 5:**
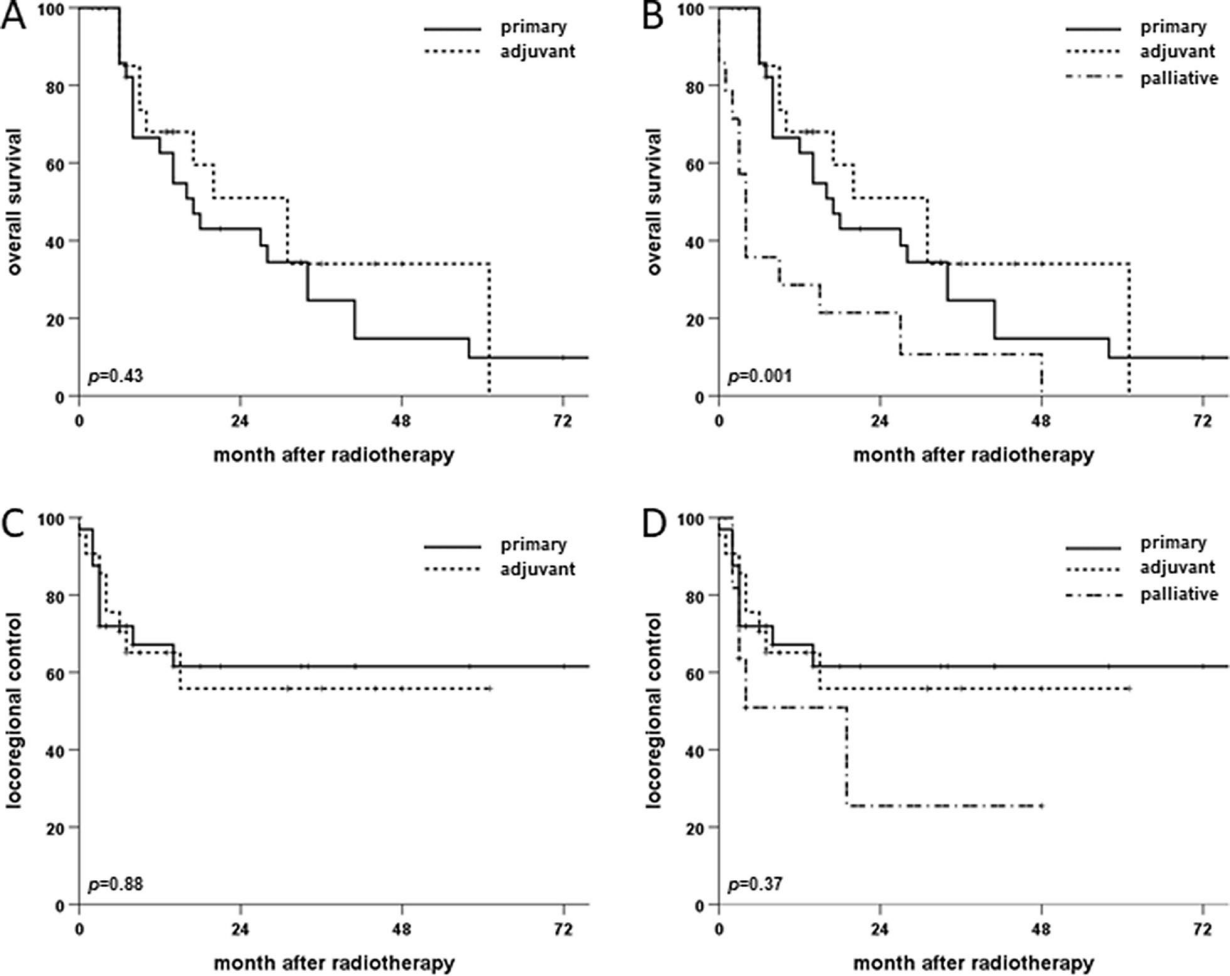
Kaplan–Meier curves showing OS (**A** and **B**) and LRC (**C** and **D**) of elderly cSCC patients (> 65 years) following primary, adjuvant or palliative radiotherapy (n = 69). *P*-values of log-rank tests are displayed

Interestingly, primary radiotherapy and resection with adjuvant radiotherapy resulted in comparable survival and LRC (*p* = 0.43 and *p* = 0.88) (Fig. 5), showing the value of adjuvant radiotherapy also for elderly patients in case of incomplete resection. Median LRC was 19 months for patients treated in palliative intend and was not reached for primary curative or adjuvant treatment, although this difference was not statistically significant (*p* = 0.37). OS after palliative radiotherapy amounted to only 11.2 months and was significantly worse than after curative treatment (*p* = 0.001).

Locoregional failure differed for various tumor localizations from no recurrence in seven tumors of the scalp to three out of four tumors of the temporal area (Additional file 2: Figure S1). Due to low case numbers statistical significance was not reached (*p* = 0.12). Incidentally, LRC was markedly better after electron beam radiotherapy with a median of 14 months in the photon cohort and the median not reached in the electron beam cohort (*p* = 0.004). This difference was likely due to a lower prevalence of nodal involvement (5% versus 28%), incomplete resection (16% versus 33%) and recurrent disease (26% versus 50%) in the electron beam cohort (Table 3).

**Table 3:** Comparison of clinical parameters for radiotherapy subgroups, primary vs. adjuvant and photon vs. electron radiotherapy in elderly cSCC patients

	Primary RT (n = 33)		Adjuvant RT (n = 22)	
	%	n	%	n
Mean age	84.7	(66–99)	80.3	(70–90)
N+	12.1	4	22.7	5
R+	–	–	63.6	14
Initial diagnosis	48.5	16	31.8	7
Local recurrence	42.4	14	45.5	10
Locoregional recurrence	9.1	3	22.7	5

	Photon RT (n = 46)		Electron RT (n = 19)	
	%	n	%	n
Mean age	83.1	(66–99)	84.3	(70–99)
N+	28.3	13	5.3	1
R+	32.6	15	15.8	3
Initial diagnosis	26.1	12	68.4	13
Local recurrence	50.0	23	26.3	5
Locoregional recurrence	23.9	11	5.3	1

In the univariate analysis, an age above 80 years (HR 2.22, CI 1.07–4.60, *p* < 0.05) and nodal disease (HR 3.68, CI 1.52–8.95, *p* < 0.01) were found to result in reduced OS, while positive resection margins showed a trend towards impaired OS (HR 5.38, CI 0.70–41.08, *p* = 0.10). In contrast, both patient performance status (HR 1.10, CI 0.60–2.00, *p* = 0.76) and comorbidity burden (HR 1.14, CI 0.63–2.05, *p* = 0.67) were found to have no prognostic influence in our cohort. In the multivariate analysis, lymph node involvement remained the only statistically significant prognostic parameter influencing OS (HR 3.73, CI 1.54–9.03, *p* < 0.01) (Table 4). In a Cox regression model for the LRC, nodal involvement was the only significant factor worsening LRC (HR 3.72, CI 1.12–12.3, *p* = 0.03). Patients treated for recurrent disease showed a trend to worse LRC (HR 1.71, CI 0.99–2.96, *p* = 0.06) (Table 4).

**Table 4:** Univariate and multivariate analysis of clinical and pathological parameters regarding OS in elderly cSCC patients receiving radiotherapy

Univariate	HR for OS	CI 95%	*p*-value
Age > 80 years	2.22	1.07–4.60	0.032
N+	3.68	1.52–8.95	0.004
R+	5.38	0.70–41.08	0.105
CCI ≥ 6	1.14	0.63–2.05	0.670
KPS ≤ 70%	1.10	0.60–2.00	0.763

Multivariate	HR for OS	CI 95%	*p*-value
Age > 80 years	1.90	0.78–4.65	0.159
N+	3.73	1.54–9.03	0.004

Univariate	HR for LRC	CI 95%	*p*-value
Age > 80 years	0.68	0.30–1.52	0.351
T stage	1.63	0.90–2.96	0.108
R+	2.22	0.28–17.8	0.454
N+	3.72	1.12–12.3	0.031
total dose (EQD2)	0.99	0.96–1.02	0.496
recurrence vs. initial diagnosis	1.71	0.99–2.96	0.058
KPS ≤ 70%	0.90	0.39–2.06	0.803

### Toxicity

Treatment-related toxicity was moderate in our cohort of elderly cSCC patients undergoing radiotherapy. Only 3 patients (4.3%) reported any higher-grade acute toxicity (CTCAE grade 3) (Tables 5, 6). 81.2% of patients (n = 56) suffered from at least one mild or moderate (CTCAE grade 1–2) adverse event, mostly dermatitis (80%), dysgeusia (17%) and xerostomia (17%). No acute grade 4 or 5 toxicities were observed.

**Table 5:** Toxicity results after radiotherapy of elderly patients with cSCC according to the Common Terminology Criteria for Adverse Events (CTCAE) v5.0

	n	%
**Acute toxicity (n = 69)**		
CTCAE 0	10	14.5
CTCAE 1–2	56	81.2
CTCAE 3	3	4.3
CTCAE ≥ 4	0	0.0
**Chronic toxicity (n = 62)**		
CTCAE 0	47	75.8
CTCAE 1	22	35.5
CTCAE 2–5	0	0.0

**Table 6:** Toxicity results consisting various radiotherapy-related adverse reactions according to the Common Terminology Criteria for Adverse Events (CTCAE) v5.0

CTCAE	0	1	2	3	4	5
**Acute (n = 69)**						
Skin toxicity	13	45	10	1	0	0
Dysphagia	62	6	0	1	0	0
Weight loss	63	4	1	0	–	–
Nausea	67	2	0	0	–	–
Mucositis	53	5	10	1	0	0
Xerostomia	57	9	3	0	–	–
Hoarseness	69	0	0	0	–	–
Dyspnea	69	0	0	0	0	0
Dysgeusia	57	11	1	–	–	–
Pain	59	5	5	0	–	–
Cytopenia	68	1	0	0	0	0
Otitis	68	0	1	0	0	0
Conjunctivitis	63	4	2	0	0	0
Infection	66	1	2	0	0	0
Hearing loss	63	5	1	0	0	0
Hyposmia	69	0	0	0	0	0
Neuropathy	68	1	0	0	0	0
Alopecia	67	2	0	0	0	0
Lymphedema	67	2	0	0	0	0
Epiphora	67	2	0	0	0	0
Vertigo	68	1	0	0	0	0
**Chronic (n = 62)**						
Skin toxicity	55	7	0	0	0	0
Dysphagia	61	1	0	0	0	0
Weight loss	62	0	0	0	–	–
Nausea	62	0	0	0	–	–
Mucositis	62	0	0	0	0	0
Xerostomia	53	9	0	0	–	–
Hoarseness	62	0	0	0	–	–
Dyspnea	62	0	0	0	0	0
Dysgeusia	59	3	0	–	–	–
Pain	60	2	0	0	–	–
Cytopenia	62	0	0	0	0	0
Renal insufficiency	62	0	0	0	0	0
Jaw and dental injuries	61	1	0	0	0	0
Neuropathy	62	0	0	0	0	0
Hyposmia	62	0	0	0	0	0
Alopecia	62	0	0	0	0	0
Hearing loss	58	4	0	0	0	0
Hyperpigmentation	57	5	0	0	0	0
Xerophthalmia	61	1	0	0	0	0
Tinnitus	60	2	0	0	0	0
Decreased vision	61	1	0	0	0	0
Lymphedema	61	1	0	0	0	0
Epiphora	61	1	0	0	0	0

Similarly, the prevalence of chronic toxicities was very low in our patient cohort. Only 22 patients (35%) experienced at least one mild chronic toxicity (CTCAE grade 1). Reported chronic toxicities were skin-related in 12 patients (19%), xerostomia in 9 patients (15%) and hearing impairments in 4 patients (6%). Importantly, no grade 2 to 5 chronic toxicities were observed.

## Discussion

In this study, we demonstrated comparably acceptable LRC rates for definitive radiotherapy and adjuvant radiotherapy after incomplete resection. Previous retrospective analyses reported higher LRC rates after definitive photon radiotherapy ranging at almost 90% [12, 21, 22]. Only the cohort of Cognetta et al. comprised a comparable patient age with a mean of 79 years. In contrast to our study, almost all patients in the studies of Grossi et al. as well as Cognetta et al. exhibited T1 tumors without nodal metastases or high-risk features, as a possible explanation for the favorable outcome. For this low-risk tumors orthovoltage techniques has been used by Grossi and Cognetta. This technique is not available at our department, and could have been used only for a small number of our patients, given the high prevalence of high-risk features. In addition, patients in these reported cohorts were referred to primary radiotherapy, whereas in our cohort, only those patients who were not eligible for surgery were enrolled to receive primary radiotherapy. It has to be noted that the evaluation of local failure is complicated by the commonly displayed field cancerization of heavily sun-damaged areas, where newly occurring tumors in close proximity can hardly be differentiated between recurrence or de-novo cancers [23].

Importantly, we did not detect differences in survival or LRC between patients treated with primary compared to adjuvant radiotherapy. Given the higher prevalence of high-risk features in the adjuvant treatment group (Table 3), this highlights the role of adjuvant treatment even for elderly patients with high-risk features such as positive resection margins, lymph node involvement or recurrent disease. A retrospective study in patients with regional metastatic cSCC from Palme et al. found significantly worse survival after primary radiotherapy compared to multimodal treatment; however, the analysis did not show any data on LRC or detailed patient characteristics [24].

Concerning adjuvant treatment, Sun et al. reported similar LRC to our dataset after surgical resection followed by radiotherapy with around 35% locoregional failures in a cohort with a median age of 71 years [25]. A further publication by Harris et al. showed an improvement of LRC and OS by adjuvant radiotherapy compared to surgery alone in patients at high risk of tumor recurrence. The reported 2-year OS of around 70% is notably higher than our results [18]. However, it should be noted that the elderly patient cohort included in their analysis was on average ten years younger, strongly supporting our finding of patient age being the predominant survival factor.

Lymph node involvement revealed itself as the strongest prognostic factor in our cohort. A significant reduction of LRC translated into a significantly reduced OS in our multivariate Cox regression model. Comparable results regarding lymph node involvement have been described by other datasets [17, 26]. Even after primary surgery followed by adjuvant radiotherapy, the results remain unsatisfactory for patients with nodal metastases, and further systemic treatment may be considered for those patients. PD-1 inhibitors like pembrolizumab and cemiplimab have shown efficacy, but response rates of 50% or less require further patient stratification [27–29]. In addition, a case series has been suggesting radiotherapy with concomitant pembrolizumab as an alternative for inoperable cSCC [30].

Regarding the importance of LRC for the prognosis of elderly cSCC patients, escalation of radiation treatment doses may provide additional benefits to improve tumor control rates. The current guideline of the ASTRO suggests a variety of conventional and hypofractionated treatment schedules with EQD2 values of up to 77.8 Gy [20]. Median radiation doses in our cohort in the curative setting were slightly lower than that. Considering the overall low toxicity rates observed in our vulnerable patient cohort, dose escalation may also be a feasible approach even for elderly cSCC patients. Due to the high rate of patients with nodal involvement and patients with high risk for nodal spread and consecutive elective nodal irradiation, the majority of our patients has been treated with normofractionated schemes to avoid excessive toxicity. On the other hand, hypofractionated schemes, as mentioned in the ASTRO guideline, could improve treatment adherence through reduced overall treatment time especially in the elderly and should be applied whenever safely feasible.

Besides lymph node involvement, age was the second strong prognostic parameter for OS in our analysis. Given the very advanced age of our patient cohort, OS values are likely due to the non-cancer mortality of our elderly patients [30, 31]. Similarly, Harris et al. reported reduced OS and a trend towards reduced LRC in patients older than 70 years treated with adjuvant radiotherapy for cSCC, thus supporting our data [18]. Carter et al. also reported a similar risk of local recurrence, metastasis and disease-specific death between younger and elderly patients, but a significantly higher risk for death of any cause for the elderly population [14].

However, it is generally accepted that the chronological age is commonly of less importance than the biological age, and therefore, other indicators are incorporated into the treatment outcome models as surrogates for biological age such as patient performance or comorbidity burden [32–34]. Unexpectedly, neither performance status nor the burden of comorbidities had a significant influence on OS in our cohort. Although KPS has shown its prognostic value in many cancer entities [35–38], the influence of the performance status on the oncological outcomes in cSCC has not been reported in other patient datasets [12, 15, 17, 25]. Our patient cohort exhibited a relatively good performance status and an overall low burden of comorbidities, especially considering the very advanced age. This may be due to the lack of critical risk factors for cSCCs that also cause significant comorbidities as described for head-and-neck squamous cell carcinomas, where smoking and cumulative alcohol intake play major roles [38, 39]. KPS and CCI are composite parameters of multiple functional and anamnestic dimensions and therefore constitute feasible surrogate parameters for physical resources and resilience. Cancer therapies like chemotherapy and extensive surgery often severely stress these resources. Radiotherapy for cSCC in our elderly cohort was generally well tolerated with few toxicities. This may be a possible explanation for the lack of influence of KPS and CCI on patient survival.

While our analysis provides insight into the population of elderly cSCC patients treated with radiotherapy and their oncologic outcome, it has certain limitations. The retrospective character may impair access to information about treatment-related toxicity data or comorbidities. Additionally, data on perineural involvement, a reported prognostic factor for decreased survival and LRC, was only available for a small number of patients in our cohort [13, 14].

In summary, our analysis of radiotherapy for cSCC of the head-and-neck region in elderly patients indicates acceptable LRC but low OS in this adversely selected cohort. The strongest prognostic factor in the multivariate analysis for OS was lymph node involvement, emphasizing careful pretherapeutic staging. Considering the local disease burden of untreated cSCCs especially for elderly patients, primary radiotherapy constitutes a feasible treatment option even for patients with very advanced age not eligible for surgery. Further prospective studies are needed to corroborate our findings.

## Supplementary Information

**Additional file 1.** Additional information regarding cSCC localizations, reasons for palliative treatment, fractionation regimens and reasons for toxicity-related discontinuation of radiotherapy.

**Additional file 2: Figure S1.** Kaplan–Meier curves for LRC of elderly cSCC patients (> 65 years) following radiotherapy sorted by tumor localization.

## Data Availability

The datasets generated and/or analysed during the current study are not publicly available due to patients information privacy but are available from the corresponding author on reasonable request.
