# Supplementary material for: The value of primary and adjuvant radiotherapy for cutaneous squamous cell carcinomas of the head-and-neck region in the elderly
Source: Radiat Oncol. 2021 Jun 12;16:105. doi: 10.1186/s13014-021-01832-3 (PMC8199417; doi:10.1186/s13014-021-01832-3)
Supplement: Supplementary file 1 — Additional file 1. Additional information regarding cSCC localizations, reasons for palliative treatment, fractionation regimens and reasons for toxicity-related discontinuation of radiotherapy. [file 13014_2021_1832_MOESM1_ESM.docx]

Supplementary Table 1:

| **cSCC localizations** | **n** |
| --- | --- |
| nose | 15 |
| ear | 14 |
| cheek | 9 |
| scalp | 7 |
| intraparotid lymph node | 7 |
| cervical lymph node | 6 |
| retroauricular | 4 |
| temporal | 4 |
| forehead | 3 |

Supplementary Table 2:

| **Reasons for palliative intended therapy (n=14)** |
| --- |
| M1 with pulmonal metastases |
| M1 with mediastinal lymph node metastases |
| Extensive nodal metastases |
| Extensive nodal metastases |
| Bleeding control |
| Bleeding control |
| Metastatic second malignancy |
| Multilocular PECs after longtime hydroxyurea exposition |
| Large tumor with meningeal infiltration |
| Very advanced age and high burden of comorbidity |
| Very advanced age and high burden of comorbidity |
| Very advanced age |
| Very advanced age |
| Patient preference |

Supplementary Table 3:

| **Frequently used Fractionation schemes** | | |
| --- | --- | --- |
| Total dose (Gy) | Fx dose (Gy) | n |
|  |  |  |
| **Total cohort** |  | 69 |
| 70 | 2 | 8 |
| 64 – 66 | 1.8 – 2.2 | 15 |
| 59.4 - 60 | 1.8 - 2 | 12 |
| 44 - 52 | 4 | 10 |
| other |  | 24 |
|  |  |  |
| **Primary RT** |  | 33 |
| 70 | 2 | 4 |
| 64 – 66 | 1.8 – 2.2 | 8 |
| 60 | 2 | 6 |
| 44 - 52 | 4 | 6 |
| other |  | 9 |
|  |  |  |
| **Adjuvant** **RT** |  | 22 |
| 64 – 66 | 1.8 – 2.2 | 6 |
| 59.4 - 60 | 1.8 - 2 | 9 |
| 44 - 52 | 4 | 3 |
| other |  | 7 |

Supplementary Table 4:

| **Reasons for toxicity-associated discontinuation of RT (n=7)** |
| --- |
| Mucositis CTCAE 2, pain CTCAE 2, fatigue CTCAE 2, boost discontinued by patient request |
| Dysphagia CTCAE 3: exiccosis, electrolyte derailment and delirium, prolonged inpatient therapy |
| Otitis externa, therefore omission of boost |
| Dermatitis CTCAE 2 and severe dementia, patient did not tolerate further RT |
| Dermatitis CTCAE 2 and mucositis CTCAE 2, omission of last fraction |
| Dermatitis CTCAE 2 and conjunctivitis, omission of last fraction |
| Dermatitis CTCAE 1, omission of last fraction |
