# Supplementary figures and images for: The value of primary and adjuvant radiotherapy for cutaneous squamous cell carcinomas of the head-and-neck region in the elderly
Source: Radiat Oncol. 2021 Jun 12;16:105. doi: 10.1186/s13014-021-01832-3 (PMC8199417; doi:10.1186/s13014-021-01832-3)

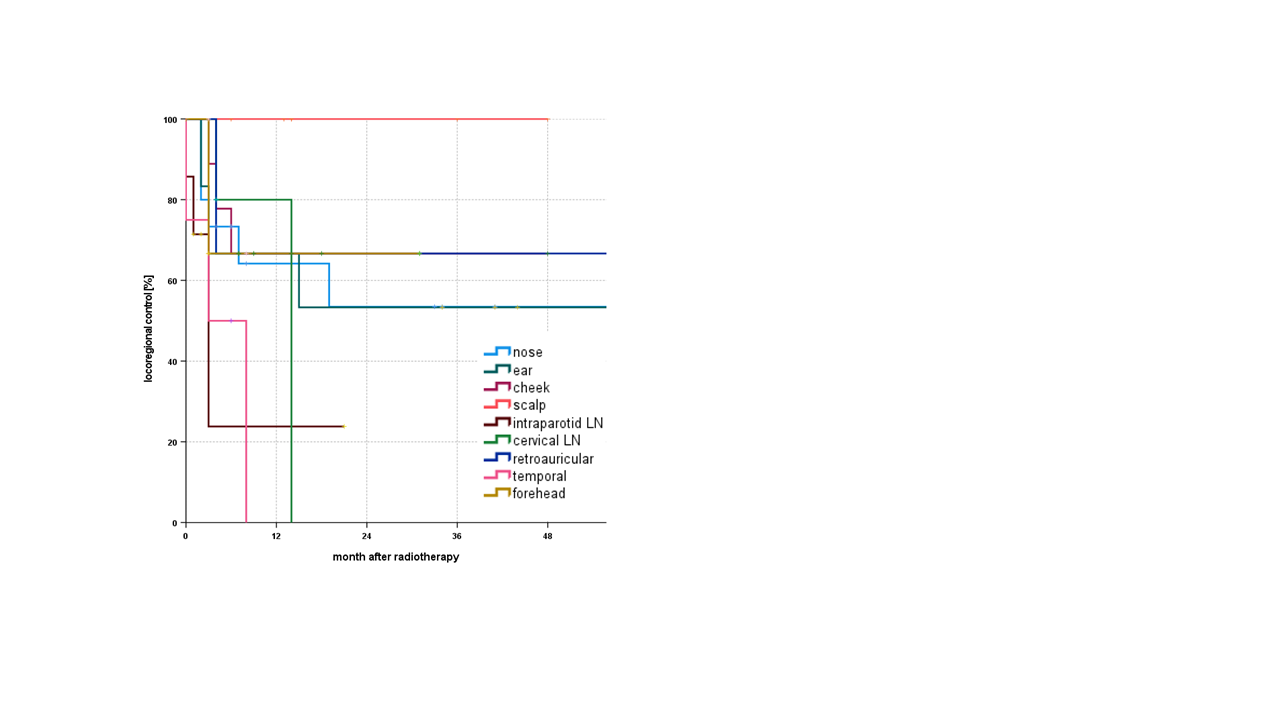

Supplement: Supplementary file 2 — Additional file 2: Figure S1. Kaplan–Meier curves for LRC of elderly cSCC patients (> 65 years) following radiotherapy sorted by tumor localization. [file 13014_2021_1832_MOESM2_ESM.tif]
